# *De novo GABRA1* variants in childhood epilepsies and the molecular subregional effects

**DOI:** 10.3389/fnmol.2023.1321090

**Published:** 2024-01-10

**Authors:** Wen-Hui Liu, Sheng Luo, Dong-Ming Zhang, Zi-Sheng Lin, Song Lan, Xin Li, Yi-Wu Shi, Tao Su, Yong-Hong Yi, Peng Zhou, Bing-Mei Li

**Affiliations:** ^1^Institute of Neuroscience and Department of Neurology of the Second Affiliated Hospital of Guangzhou Medical University, Key Laboratory of Neurogenetics and Channelopathies of Guangdong Province and the Ministry of Education of China, Guangzhou, China; ^2^Department of Neurology, Maoming People’s Hospital, Maoming, China; ^3^Department of Pediatrics, The Second Hospital, Cheeloo College of Medicine, Shandong University, Jinan, China

**Keywords:** *GABRA1* gene, epilepsy, variants, GABA_*A*_R, molecular subregional effect

## Abstract

**Background:**

The *GABRA1* gene, encoding the GABR_*A*_R subunit α1, plays vital roles in inhibitory neurons. Previously, the *GABRA1* gene has been identified to be associated with developmental and epileptic encephalopathy (DEE) and idiopathic generalized epilepsy (IGE). This study aims to explore the phenotypic spectrum of *GABRA1* and molecular subregional effect analysis.

**Methods:**

Trios-based whole-exome sequencing was performed in patients with epilepsy. Previously reported *GABRA1* mutations were systematically reviewed to analyze the molecular subregional effects.

**Results:**

*De novo GABRA1* mutations were identified in six unrelated patients with heterogeneous epilepsy, including three missense mutations (p.His83Asn, p.Val207Phe, and p.Arg214Cys) and one frameshift mutation (p.Thr453Hisfs*47). The two missense mutations, p.His83Asn and p.Val207Phe, were predicted to decrease the protein stability but no hydrogen bond alteration, with which the two patients also presented with mild genetic epilepsy with febrile seizures plus and achieved seizure-free status by monotherapy. The missense variant p.Arg214Cys was predicted to decrease protein stability and destroy hydrogen bonds with surrounding residues, which was recurrently identified in three cases with severe DEE. The frameshift variant p.Thr453Hisfs*47 was located in the last fifth residue of the C-terminus and caused an extension of 47 amino acids, with which the patients presented with moderated epilepsy with generalized tonic-clonic seizures alone (GTCA) but achieved seizure-free status by four drugs. The four variants were not presented in gnomAD and were evaluated as “pathogenic/likely pathogenic” according to ACMG criteria. Analysis of all reported cases indicated that patients with mutations in the N-terminal extracellular region presented a significantly higher percentage of FS and DEE, and the patients with variants in the transmembrane region presented earlier seizure onset ages.

**Significance:**

This study suggested that *GABRA1* variants were potentially associated with a spectrum of epilepsies, including EFS+, DEE, and GTCA. Phenotypic severity may be associated with the damaging effect of variants. The molecular subregional effects help in understanding the underlying mechanism of phenotypic variation.

## 1 Introduction

Gamma-aminobutyric acid (GABA) is the main inhibitory neurotransmitter in the human brain (Sanacora et al., 2003). Functional GABA regulates neuronal excitability by interacting with neuronal receptors, including GABA A-type receptors (GABA_*A*_R) and GABA B-type receptors (GABA_*B*_R). The GABA_*A*_R, a ligand-gated ion channel, is the primary inhibitory receptor of the central nervous system (Hannan et al., 2020; Papasergi-Scott et al., 2020; Feng et al., 2022). GABA_*A*_R is composed of various subunits, with various combinations. In the central nervous system, the most common components of GABA_*A*_R are two α subunits, two β subunits, and one γ subunit, forming a pentameric arrangement of αββαγ (Bai et al., 2019; Tomita, 2019). Dysregulation of GABA_*A*_R is the core mechanism of diverse central nervous disorders, especially epilepsies (Maljevic et al., 2019; Fu et al., 2022). Genes encoding the GABA_*A*_R subunit have been identified as causative genes of idiopathic generalized epilepsy (IGE) and/or developmental and epileptic encephalopathy (DEE), including *GABRA1*, *GABRA2*, *GABRB3*, *GABRA5*, *GABRB1*, *GABRB2*, *GABRB3*, and *GABRG2* (OMIM, database).^1^

The *GABRA1* gene (OMIM*137160), located at chromosomal locus 5q34, encodes the GABR_*A*_R subunit α 1. It forms the γ_2_β_2_α_1_β_2_α_1_ pentamer with GABR_*A*_R β2 subunits and GABR_*A*_R γ2 subunit, which is the most prevalent and functionally significant GABA*^A^*R subtype in the brain (Huang et al., 2001; Sanacora et al., 2003; Maljevic et al., 2019). The *GABRA1* gene is highly expressed in the developing brain, especially in the frontal cortex, anterior cingulate cortex, hippocampus, and hypothalamus (GTEx database).^2^ In mice, knockout of *gabra1* led to spontaneous seizures and pre-weaning lethality with incomplete penetrance (Vicini et al., 2001). In humans, the *GABRA1* gene has been identified as the causative gene of developmental and epileptic encephalopathy-19 (DEE-19, OMIM* 615744) and the susceptibility genes of childhood absence epilepsy-4 (ECA-4, OMIM* 611136) and juvenile myoclonic epilepsy-5 (JME-5, OMIM* 611136). However, the phenotypic spectrum and genotype-phenotype correlation of *GABRA1* remain undetermined.

In the present study, we performed *GABRA1* trio-based whole exome sequencing (WES) in patients with epilepsy without acquired cause. Four *de novo* mutations in the *GABRA1* variants were identified in six unrelated cases, including two with epilepsy with febrile seizures plus (EFS +), three with developmental and epileptic encephalopathy (DEE), and one with epilepsy with generalized tonic-clonic seizures alone (GTCA). To analyze the molecular subregional effects of *GABRA1*, we systematically reviewed all reported disease-causing variants and their associated phenotypes.

## 2 Materials and methods

### 2.1 Patients

The patients of the study were recruited through the Epilepsy Center of the Second Affiliated Hospital of Guangzhou Medical University, the Second Hospital of Shandong University, and the Maoming People’s Hospital. Clinical data of the individuals were collected, including present age, gender, age at seizure onset, seizure course, detailed family history, general and neurological examination results, and effective antiseizure medications (ASMs). Magnetic resonance imaging (MRI) scans were conducted to detect structural abnormalities within the brain. A long-term (24-h) video electroencephalogram (EEG) was performed including an open–close eyes test, intermittent photic stimulation, hyperventilation, and sleeping recording. Epileptic seizures and epilepsies were diagnosed based on the classification guidelines developed by the Commission on Classification and Terminology of the Wirrell et al. (2022).

This study was approved by the ethics committee of The Second Affiliated Hospital of Guangzhou Medical University (2020-h5-49), and written informed consent was obtained from all patients and their parents.

### 2.2 WES and genetics analysis

Blood samples were collected from all probands, parents, and available family members to extract genomic DNA and perform trio-based whole-exome sequencing using the Illumina HiSeq 2000 system (BGI, Shenzhen, China). Detailed sequence procedures were described in previous studies (Wang et al., 2018; Cai et al., 2019; Shi et al., 2019).

To identify potential pathogenic variants, each case was analyzed as previously described (Chen et al., 2022; Li et al., 2022; Luo et al., 2022). Initially, polymorphic variants with a minor allele frequency (MAF) ≥ 0.005 in the gnomAD database were excluded from further analysis.^3^ Potentially disease-causing variants, including frameshift, nonsense, canonical splice site, initiation codon, in-frame, and missense variants were retained for further study. We screened *GABRA1* mutations with potentially pathogenic inheritance patterns, including *de novo*, cosegregated, homozygous, and compound heterozygous, which demonstrate the genetic difference between the affected child and the asymptomatic parents in a given family (trio). All *GABRA1* mutations identified in this study were annotated into the reference transcript NM_ 000806.5.

### 2.3 Functional prediction and bioinformatic analysis

The consequences of missense variants were predicted by multiple *in silico* tools with detailed scores acquired from the VarCards database (Li et al., 2018),^4^ including SIFT, VEST3, MetaSVM, MetaLR, M-CAP, CADD, DANN, Eigen, Fathmm-MKL, and REVEL. The Varsite web server was utilized to examine the hydrophobicity alterations of variants (Laskowski et al., 2020).^5^ MetaDome analyses the mutation tolerance at each position in a human protein. The conservation of mutated positions was evaluated through sequence alignment across different species.^6^

### 2.4 Protein modeling

The structural impact of potential missense variants was evaluated by conducting protein modeling using the Iterative Threading ASSEmbly Refinement software (I-TASSER) (Yang and Zhang, 2015).^7^ The visualization and analysis of the protein’s three-dimensional structure were conducted using the PyMOL Molecular Graphics System (Version 2.3.2). To estimate the impact of protein stability of variants, the I-Mutant server was used to predict the free energy change value (DDG, kcal/mol) (Capriotti et al., 2005).^8^

### 2.5 Review of previously reported variants and genotype-phenotype analysis

To evaluate the relationship between genotype and phenotype, we conducted a comprehensive review of all previously reported *GABRA1* mutations and associated clinical information via the PubMed database^9^ and the Human Gene Mutation Database (HGMD)^10^ until August 2023, likely our previously studies (Yan et al., 2022; Luo et al., 2023). Variants with undefined origination or unexplained origination for the occurrence of genetic diseases were excluded. The variants were visualized in the protein diagram. The number of variants with varied phenotypes in distinct functional domains was compared by Fisher’s exact test, to analyze the molecular subregional effects.

### 2.6 Statistical analysis

The statistical analyses were performed utilizing R software (version 4.0.3). Fisher’s exact test was used to evaluate the frequencies of *GABRA1* mutations in the case-cohort and the control populations (2015). A *P-*value < 0.05 was considered to be statistically significant.

## 3 Results

### 3.1 Identification of *GABRA1* variants

**FIGURE 1:**
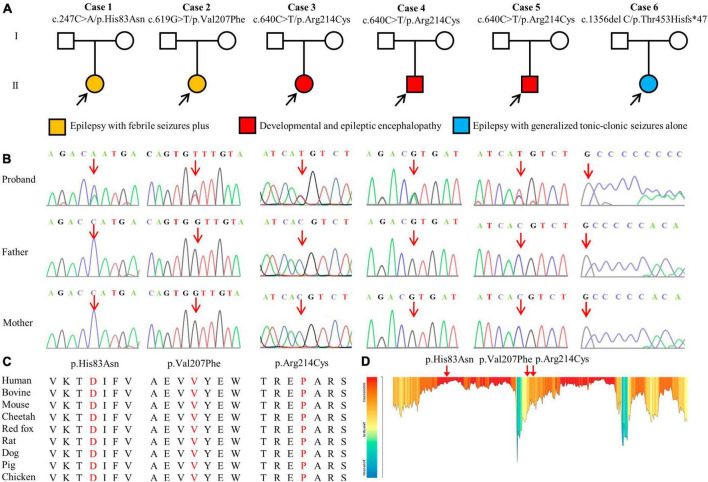
Genetic data of patients with *GABRA1* mutations. **(A)** Pedigrees of the six *de novo* mutations and their corresponding phenotypes. **(B)** DNA sequence chromatogram of the *GABRA1* mutations. Arrows indicate the positions of the mutations. **(C)** The amino acid sequence alignment of the three missense mutations shows that the residues are highly conserved across mammals. **(D)** The tolerance of three missense mutations of *GABRA1* predicted by the MetaDome algorithm.

A total of four *GABRA1* mutations were identified in six unrelated cases, including three missense mutations (c.247C > A/p.His83Asn, c.619G > T/p.Val207Phr, and c.640C > T/p.Arg214Cys) and one frameshift mutation (c.1356delC/p.Thr453Hisfs*47) (Figures 1A, B; Table 1). The mutations c.640C > T/p.Arg214Cys was recurrently identified in three cases, including cases 3, 4, and 5. The six variants were all of *de novo*, consistent with the autosomal dominant inheritance pattern.

**TABLE 1:** Clinical features of the individuals with *GABRA1* mutations.

Case	Variants (NM_000806.5)	Gender	Age	Onset age of seizure	Seizure course	Prognosis	Effective ASMs	EEG	Brain MRI	Development	Diagnosis
Case 1	c.247C > A/p.His83Asn	F	4yr	11mo	GTCS 7-10 times/yr, mostly febrile-related	Seizure-free for 6 months	PER	Background: normal. Interictal: spike-slow and poly-spike-slow waves in the bilateral central with predominance in the right (at the age of 3 years)	Normal	Normal	EFS +
Case 2	c.619G > T/p.Val207Phe	F	14yr	2yr	FS 2–3 times/yr. (<6yr) CPS 5–6 times/yr.	Seizure-free for 2 years	LEV	Background: normal. Interictal: generalized sharp-slow and spike-slow waves and multifocal spike-slow (at the age of 10 years)	Normal	Normal	EFS +
Case 3	c.640C > T/p.Arg214Cys	F	19yr	6mo	CPS and GTCS 2 times/yr, mostly febrile-related	Seizure-free for 12 years	VPA, TMP	Background: normal. Interictal: sharp and spike waves in bilateral central, partial, and posterior-temporal, and generalized spike-slow waves (at the age of 16 years)	Normal	Intellectual disability	DEE (DS-like)
Case 4	c.640C > T/p.Arg214Cys	M	6yr	7mo	fbTCS 1–2 times/mo.	Seizure-free for 1 years	VPA, LTG, LEV, TPM	Background: diffuse slow waves. Interictal: the right temporal δ waves and left occipital sharp waves (at the age of 3.5 years)	The right hippocampus smaller than the left	Intellectual disability and speech delay	DEE
Case 5	c.640C > T/p.Arg214Cys	M	8yr	1y r	CPS, fbTCS, and GTCS, 6–7 times/yr. mostly febrile-related	Seizure-free for 2.5 years	VPA, LEV	Background: diffuse slow waves. Interictal: generalized sharp-slow and spike-slow waves (at the age of 7 years)	The white matter abnormalities around the bilateral lateral ventricle	Severe intellectual disability (IQ 29) and speech delay	DEE (DS-like)
Case 6	c.1356delC/p.Thr453Hisfs*47	F	19yr	11yr	GTCS, 1–4 times/yr.	Seizure-free for 6 months	LTG, VPA, LEV, CLB	Background: normal. Interictal: generalized 3–6 Hz spike-slow wave (at the age of 16 years)	Normal	Normal	GTCA

ASMs, antiseizure medications; CLB, clobazam; CPS, complex partial seizures; F, female; M, male; DEE, developmental and epileptic encephalopathy; EEG, electroencephalography; FS, febrile seizures; fbTCS, focal to bilateral tonic clonic seizure; EFS + , epilepsy with febrile seizures plus; GTCA, epilepsy with generalized tonic?clonic seizures alone; GTCS, generalized tonic?clonic seizures; LTG: lamotrigine; LEV, levetiracetam; mo, month; MRI, magnetic resonance imaging; MAF, minor allele frequency; PE, partial epilepsy; PER, perampanel; TPM, topiramate; VPA, valproate; yr, year.

The four *de novo* variants were not present in the population of the gnomAD database. Amino acid sequence alignment suggested that the variants were located in residues with high conservation across mammals (Figure 1C). The three variants were located in residues that are intolerant of missense variants, according to the MataDome prediction (Figure 1D). The missense mutations were all predicted to be “conserved” or “damaging” by multiple *in silico* algorithms. According to the ACMG guidelines, the frameshift mutation p. Thr453Hisfs*47 and the recurrently identified missense mutation p. Arg214Cys were evaluated as “pathogenic,” while the other two missense mutations, p.His83Asn and p.Val207Phe, were rated as “likely pathogenic” (Table 2).

**TABLE 2:** Genetic characteristics of *GABRA1* mutations.

Case numb.	cDNA change (NM_000806.5)	Protein change	Inheritance	MAF	SIFT	VEST3	MetaSVM	MetaLR	M-CAP	CADD	DANN	Eigen	Fathmm- MKL	REVEL	ACMG
1	c.247C > A	p.His83Asn	*De novo*	-/-	D (0.018)	T (0.375)	T (–0.57)	T (0.268)	D (0.063)	D (23.9)	T (0.685)	D (0.175)	D (0.930)	P (0.814)	LP (PS2 + PM2 + PP3)
2	C.619G > T	p.Val207Phe	*De novo*	-/-	D (0.01)	D (0.781)	D (0.372)	D (0.617)	D (0.245)	D (32)	D (0.997)	D (0.823)	D (0.995)	D (0.675)	LP (PS2 + PM2 + PP3)
3, 4, 5	C.640C > T	p.Arg214Cys	*De novo*	-/-	D (0.0)	D (0.802)	D (0.506)	D (0.698)	D (0.362)	D (35)	D (0.999)	D (0.706)	D (0.930)	D (0.701)	Pathogenic (PS1 + PS2 + PM2 + PP3)
6	c.1356delC	p.Thr453Hisfs*47	*De novo*	-/-	–	–	–	–	–	–	–	–	–	–	Pathogenic (PVS1 + PS2 + PM2)

D, disease-causing; B, benign; C, conserved; CADD, combined annotation dependent depletion; D, damaging; fitCons, the fitness consequences of functional annotation; MAF, minor allele frequency from gnomAD (controls); MAF-EAS, minor allele frequency from gnomAD (controls)-East Asian population; NA, not available; NC, non-conserved; P, polymorphism; PP2_Var, polyphen2_HVAR; T, tolerable; LP: Likely pathogenic.

### 3.2 Clinical features

Heterogeneous epilepsies were present in the six cases with *GABRA1* mutations, including two with EFS+, three with DEE, and one with GTCA. Detailed clinical information was summarized in Table 1.

**FIGURE 2:**
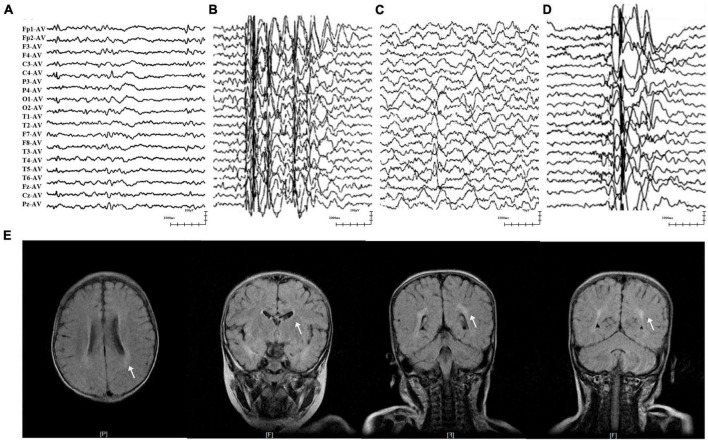
Representative EEG and MRI of patients with *GABRA1* variants. **(A)** Interictal EEG of case 1 showed multifocal waves at the age of 3 years. **(B)** Interictal EEG of case 2 showed generalized spike-slow waves at the age of 10 years. **(C)** Interictal EEG of case 4 showed right temporal slow waves at the age of 3.5 years. **(D)** Interictal EEG of case 5 showed generalized spike-slow waves and irregular 0.5–3 slow activity bursts 0.5–2 s during sleep. **(E)** The MRI of case 5 showed an abnormal white matter signal around the posterior horn of the lateral ventricle.

The two patients in cases 1 and 2 were diagnosed with EFS+, in which variants p.His83Asn and p.Val207Phe were identified, respectively. The two patients initially presented with febrile seizures (FS) featuring generalized tonic-clonic seizures (GTCS) at the age of 6 years old and subsequently afebrile GTCS. Seizures in the two patients were infrequent (yearly) and were controlled by monotherapy with perampanel (PER) or levetiracetam (LEV). Their interictal EEG recorded epileptic discharges, manifesting multifocal and generalized discharges in the Rolandic region (Figures 2A, B). The brain MRI scans were normal. The two patients showed normal neurodevelopment.

The three patients with the identical variant p.Arg214Cys were all diagnosed with DEE, including two who presented with Dravet-like syndrome (Cases 3 and 5). The three patients all presented infancy-onset seizures with multiple seizure types, including GTCS, focal to bilateral tonic-clonic seizure (fbTCS), and focal seizure (Fos). Seizures in cases 3 and 5 were mostly febrile-related. Seizure-free status in the three patients was achieved by combined treatment including valproate (VPA). Their EEG recorded epileptic discharge with features of DEE, including interictal multifocal, bilateral, and/or generalized discharges and disuse slow waves in the background during the waking stage (Figures 2C, D). Brain MRI scans detected structural abnormalities, including a smaller right hippocampus in case 4 and white matter abnormalities around the bilateral lateral ventricle in case 5 (Figure 2E). The three patients all exhibited intellectual disability (ID), and two patients (cases 4 and 5) also presented speech delays.

The patient in case 6, who had the frameshift mutation p.Thr453Hisfs*47, was diagnosed with GTCA. She exhibited yearly GTCS since eleven-years-old. Seizure-free status was achieved for 6 months after treatment with four ASMs, including VPA, LEV, lamotrigine (LTG), and clobazam (CLB). EEG recording showed generalized 3–6 Hz spike-slow waves. Brain MRIs and neurodevelopment of the patient were normal.

### 3.3 Molecular alteration

**FIGURE 3:**
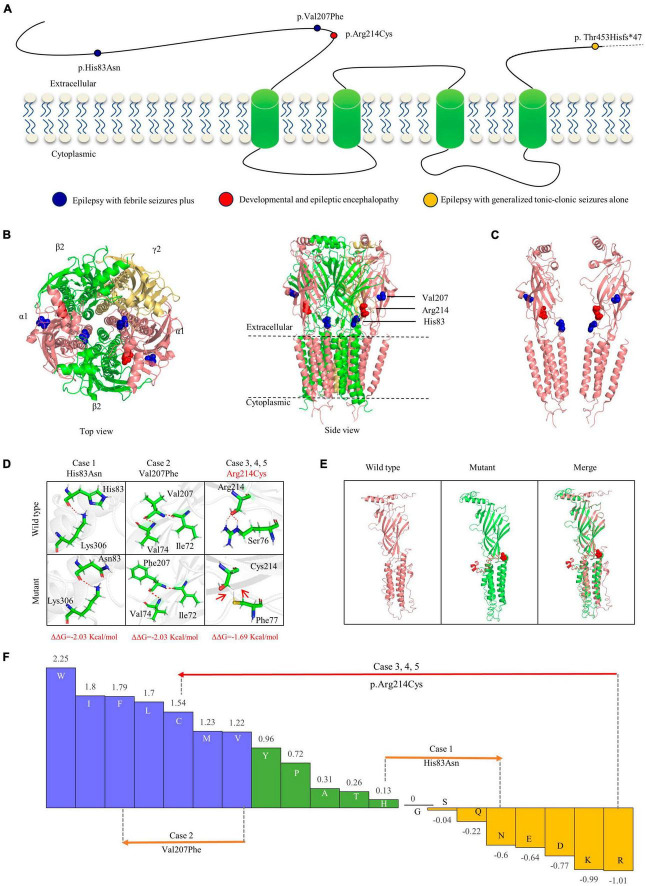
Schematic illustration of mutations and interactions with surrounding amino acids. **(A)** Schematic diagram of the GABRA1 protein and the localization of the *GABRA1* mutations identified in the study. **(B)** The 3D structural model of GABA_*A*_R γ_2_β_2_α_1_β_2_α_2_ and the location of the *GABRA1* mutations. **(C)** The location of the three missense mutations in the GABRA1 protein. **(D)** Hydrogen bond changes and DDG values of the three missense mutations. The red dotted line represents hydrogen bonds. **(E)** The structural alteration of the frameshift mutation. **(F)** Fauchère and Pliska hydrophobicity scale exhibited the hydrophobicity of 20 amino acids. Abscissa: from left to right, hydrophobicity gradually decreased. Blue amino acids are hydrophobic, green amino acids are neutral, and yellow amino acids are hydrophilic. Amino acids with high positive values are more hydrophobic, whereas amino acids with low negative values are more hydrophilic.

The GABA receptor subunit alpha1 encoded by *GABRA1* consists of one N-terminal extracellular domain, four transmembrane domains, three linkers between transmembrane domains, and one C-terminal extracellular domain (Macdonald et al., 2010). The three missense mutations identified in the present study were all located in the N-terminus extracellular domain, while the frameshift mutation was located in the latest fourth residue of the C-terminus extracellular domain (Figure 3A).

Protein modeling and single point mutation free energy change prediction were utilized to assess the structural impact of the identified mutations. The two missense mutations (p.His83Asn and p.Val207Phe) of the cases with mild EFS + were predicted to decrease the protein stability with ΔΔG >> 0.5 Kcal/mol but no hydrogen bond alteration (Figures 3B–E). The missense mutation p.Arg214Cys, which was recurrently identified in three severe DEE cases, was not only predicted to decrease protein stability but also destroy hydrogen bonds with surrounding residues. The frameshift mutation p.Thr453Hisfs*47 identified in the case with moderate GTCA was located in the last fifth residue of the C-terminus, causing an extension of 47 amino acids. The mutant protein was also predicted to cause abnormal spatial folding (Figure 3F).

### 3.4 Clinical and genetic characteristics analysis of available *GABRA1* mutations

**FIGURE 4:**
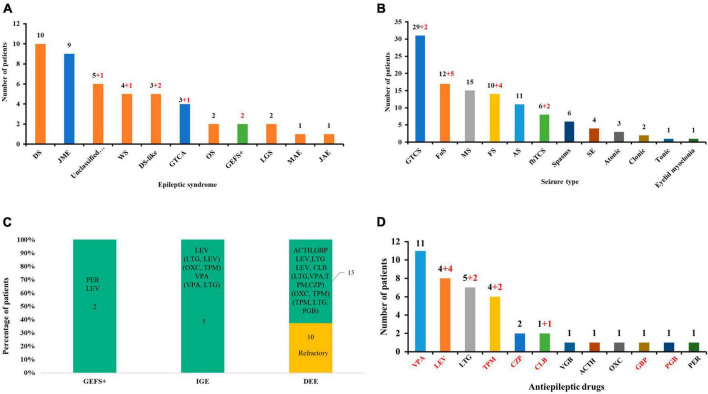
Clinical and genetic characteristics analysis of available *GABRA1* mutations. **(A)** The epileptic syndrome of patients with *GABRA1* variants. Red numbers indicate data from this study. The red column represents DEEs; the blue column represents IGEs; and the green column represents EFS+. **(B)** The seizure types of patients with *GABRA1* variants. Red numbers indicate data from this study. **(C)** The stacked bar chart of the drug response of the patients with different phenotypes. The orange colors show that the percentage of patients was refractory, and the green colors show the percentage of patients with a positive response to drugs. **(D)** The effective ASMs used in patients with *GABRA1* variants. Red numbers indicate data from this study.

A total of 25 *GABRA1* mutations with explainable origins for the occurrence of genetic diseases have been identified in 47 patients (36 cases) so far, including two novel missense and the first frameshift mutation from the present study (Supplementary Table 1). The patients presented with heterogeneous epilepsies, including DEEs, IGEs, and EFS + (Figure 4A). Variable seizure types were exhibited in those patients, including GTCS, Fos, myoclonic seizures, FS, absence, fbTCS, spasms, status epilepticus, atonic, clonic, eyelid myoclonia, and tonic seizures. GTCS, Fos, and myoclonic seizures were the most common seizure types (Figure 4B). Distinct outcomes and drug responses were also presented in these patients, including ten patients with refractory seizures, eleven patients achieving seizure-free status, and six patients with seizure remission (Figure 4C). VPA, LEV, and LTG were the most commonly used drugs in patients with a positive drug response (Figure 4D).

### 3.5 Molecular subregional effects analysis

**FIGURE 5:**
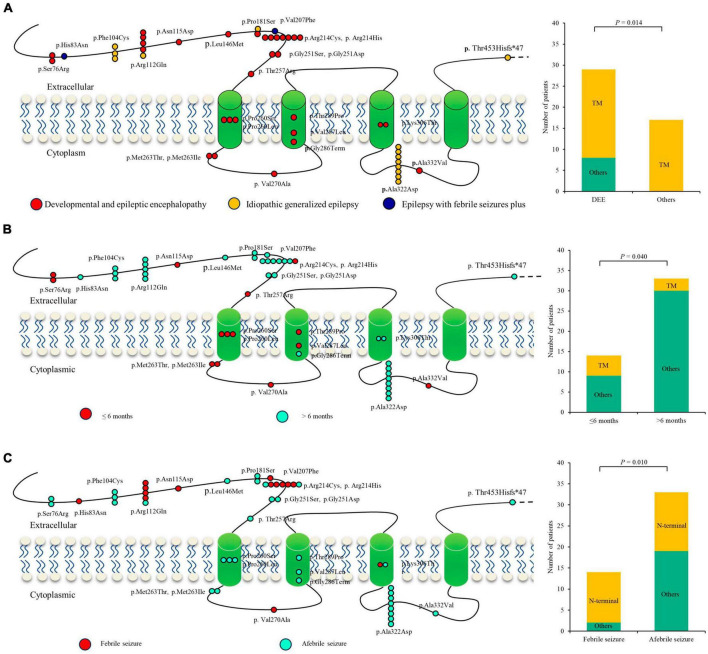
Molecular subregional effects analysis of *GABRA1* mutations. **(A)** The locations of *GABRA1* variants associated with various epilepsy syndromes, including DEEs, IGEs, and EFS+. The patients with variants in the transmembrane region all presented with severe DDE, showing a significantly higher percentage of DEE than those with mutations in other regions. TM, transmember. **(B)** The locations of *GABRA1* variants associated with varied onset ages of seizures, including early onset (younger than 6 months) and late onset (older than 6 months). The patients with variants in the transmembrane region also exhibited earlier seizure onset ages (less than 6 months old) than those with variants in other regions. **(C)** The locations of *GABRA1* variants associated with febrile seizure (FS) and afebrile seizure. The patients with mutations in the N-terminal extracellular region presented a significantly higher percentage of FS than those with mutations in other regions.

A previous study indicated that phenotypic heterogeneity was associated with molecular subregional effects (Liao et al., 2022). The location of variants and associated phenotypes were thus visualized for analysis. It is suggested that the patients with variants in the transmembrane region all presented with severe DDE, showing a significantly higher percentage of DEE than those with mutations in other regions (Figure 5A). The patients with variants in the transmembrane region also exhibited earlier seizure onset ages (less than 6 months old) than those with variants in other regions (*P* = 4.0 × 10^–2^) (Figure 5B). The patients with mutations in the N-terminal extracellular region presented a significantly higher percentage of FS than those with mutations in other regions (*P* = 1.0 × 10^–2^) (Figure 5C). These findings suggested a potential molecular subregional effect.

## 4 Discussions

In this study, we identified novel *GABRA1* variants in six unrelated cases, including one frameshift and three missense mutations. The mutations were of *de novo*, had no frequencies in controls, and were predicted to alter protein stability. According to the ACMG criteria, the four variants were all evaluated to be “pathogenic” or “likely pathogenic.” The six patients presented with heterogeneous epilepsies, including two with EFS+, three with DEE, and one with GTCA. Analysis of all reported cases indicated that patients with mutations in the N-terminal extracellular region presented a significantly higher percentage of FS, and patients with variants in the transmembrane region presented earlier seizure onset ages, indicating a potential molecular subregional effect. This study suggested that *GABRA1* is associated with a spectrum of epilepsies, including severe DEE, IGEs, and EFS+. The molecular subregional effects analysis provided novel insight into the phenotypic heterogeneity of *GABRA1*.

The *GABRA1* gene is highly conserved across various species, including zebrafish, mice, and humans. In zebrafish, knockout of *GABRA1* causes epileptic seizures triggered by light (Samarut et al., 2018). In mice, knockout of *GABRA1* led to spontaneous seizures and pre-weaning lethality with incomplete penetrance (Vicini et al., 2001). In humans, genomic data reveal that *GABRA1* is intolerant to loss-of-function (LOF) variants with the pLI (the probability of being LOF intolerant) index of 0.91 and LOEUF (LOF observed/expected upper bound fraction) index of 0.91 (Karczewski et al., 2020). In this study, we identified one frameshift and three missense mutations of *GABRA1* in six unrelated cases. The frameshift mutation (p.Thr453Hisfs*47) was predicted to cause a truncated protein, which is associated with LOF. The three missense variants were all predicted to be damaged by multiple *in silico* tools and decrease protein stability, which may also be associated with LOF. These findings suggested that LOF may be the epileptogenic mechanism of *GABRA1.*

Abnormalities of GABA_*A*_R were one of the most essential mechanisms of the occurrence of multiple epilepsies, particularly IGEs, DEEs, and EFS+. The GABR_*A*_R subunit α1 encoded by the *GABRA1* gene is the core subunit of the GABA_*A*_R γ_2_β_2_α_1_β_2_α_1_ pentamer, the most prevalent and functionally significant subtype in the brain. Previously, the *GABRA1* gene has been identified as one of the causative genes of DEE and the susceptibility gene of IGEs, including ECA-4 and EJM-5. In this study, we identified six novel cases with *GABRA1* mutations, including two cases with EFS + and one case with the IGE subtype of GTCA, expanding the phenotypic spectrum of *GABRA1*.

The two missense variants identified in cases 1 and 2 were predicted to decrease the protein stability but no hydrogen binding alteration, indicating mild damaging effects; the two cases exhibited the mild phenotype of EFS + and achieved seizure-free status by monotherapy. The variant p.Arg214Cys recurrently identified in cases 3, 4, and 5 was predicted to destroy hydrogen bonds with surrounding residues and decrease the protein stability, suggesting a severely damaging effect; the three patients also presented with the severe phenotype of DEE. The frameshift variant p.Thr453Hisfs*47 was located in the C-terminus, which only led to a frameshift of four amino acids and an additional extension of 47 amino acids and may reflect moderate damaging effects; the patient also exhibited a moderate phenotype manifesting mild GTCA but achieving seizure-free status by four drugs. This finding suggests that phenotypic severity is possibly associated with the damaging effects of variants.

Previous studies have indicated that molecular subregional effects may also be associated with phenotypic heterogeneity (Liao et al., 2022). This study suggested that the patients with variants in the transmembrane region tended to present with severe DEE and earlier seizure onset ages, while patients with variants in the N-terminal extracellular region appeared to exhibit FS. These findings suggested a potential molecular subregional effect, which warrants a larger case-cohort study to further determine.

The three patients (Cases 3, 4, and 5) with the identical variant p.Arg214Cys presented with comparable phenotypic severity but subtle phenotypic variation, including two with Dravet-like syndrome and one with DEE without FS. The three previously reported cases with the identical variant p.Arg214Cys also presented phenotypic heterogeneity, including two with Dravet syndrome and one with Lennox-Gastaut syndrome (Johannesen et al., 2016; Cai et al., 2019). An analogous situation was also presented in other hotspot mutations, including p.Ser76Arg, p.Phe104Cys, p.Arg112Gln, p.Pro181Ser, p.Arg214His, p.Arg214Cys, p.Pro260Leu, and p.Lys306Thr (Supplementary Table 1). It is unknown whether modifier genes or polygenic interactions also partially contribute to phenotypic heterogeneity.

In this study, seizure-free status was achieved in all patients, in which five of six patients were treated with VPA and/or LEV. Analysis of previously reported patients with *GABRA1* mutations also showed that VPA and LEV were the most commonly used ASMs in patients with positive drug responses. Previous studies suggested that the GABAergic system was one of the main targets of VPA and LEV, which may be a possible explanation for the positive effect of the two drugs in patients with *GABRA1* mutations (Deshpande and Delorenzo, 2014; Romoli et al., 2019). Early genetic diagnosis may help in the clinical management of patients with *GABRA1* mutations.

In conclusion, this study suggested that *GABRA1* variants were potentially associated with a spectrum of epilepsies, including EFS+, DEE, and GTCA. Phenotypic severity may be associated with the damaging effect of variants. The molecular subregional effects help in understanding the underlying mechanism of phenotypic variation. Early use of VPA, LEV, and/or LTG may be the optimal treatment option for patients with *GABRA1* mutations if early genetic diagnosis is acquired.

## Data availability statement

The data presented in the study are deposited in the NCBI-genebank repository, accession number OR818666-OR818683.

## Ethics statement

The studies involving humans were approved by the Ethics Committee of the Second Affiliated Hospital of Guangzhou Medical University. The studies were conducted in accordance with the local legislation and institutional requirements. Written informed consent for participation in this study was provided by the participants’ legal guardians/next of kin.

## Author contributions

W-HL: Writing—original draft. SLuo: Conceptualization, Formal analysis, Supervision, Writing—review and editing. D-MZ: Resources, Validation, Writing—review and editing. Z-SL: Writing—review and editing. SLan: Writing—review and editing. XL: Writing—review and editing. Y-WS: Writing—review and editing. TS: Writing—review and editing. Y-HY: Writing—review and editing. PZ: Writing—review and editing. B-ML: Writing—review and editing.

## Footnotes

1
https://omim.org/

2
https://www.gtexportal.org/

3
https://gnomad.broadinstitute.org/

4
http://www.genemed.tech/varcards/

5
www.ebi.ac.uk/thornton-srv/databases/cgi-bin/VarSite/GetPage.pl?home=TRUE

6
https://stuart.radboudumc.nl/metadome/

7
https://zhanglab.ccmb.med.umich.edu/I-TASSER/

8
https://folding.biofold.org/i-mutant/i-mutant2.0.html

9
http://www.ncbi.nlm.nih.gov/pubmed/

10
http://www.hgmd.cf.ac.uk/ac/index.php
