# Supplementary material for: De novo GABRA1 variants in childhood epilepsies and the molecular subregional effects
Source: Front Mol Neurosci. 2024 Jan 10;16:1321090. doi: 10.3389/fnmol.2023.1321090 (PMC10806124; doi:10.3389/fnmol.2023.1321090)
Supplement: Supplementary file 1 [file Table_1.DOCX]

**Table S1.** Characteristics of previously reported cases with *GABRA1* mutations.

| **Case** | **Gender** | **Variants** | **MAF** | **Originated** | **Onset-age** | **Seizure type** | **Frequency of seizure** | **Therapy** | **Neurodevelopment** | **EEG** | **MRI** | **Diagnosis** | **Ref** |
| --- | --- | --- | --- | --- | --- | --- | --- | --- | --- | --- | --- | --- | --- |
| 1 | M | c.226A>C/p.Ser76Arg | *-/-* | *DN* | 5 mo | FoS, SE, fbTCS, GTCS | NA | Refractory | Severe GDD, speech delay, and behavioral problems | Blateral or multifocal paroxysmal activity | Normal | DEE  (DS-like) | [2] |
| 2 | F | c.226A>C/p.Ser76Arg | *-/-* | *DN* | 6 mo | GTCS, MS, AS | NA | Refractory | Severe GDD, speech delay, and autistic features | Generalized spike-waves, intermittent photic stimulation response | Normal | DEE  (DS) | [2] |
| 3-1 | M | c.311G>A/p.Phe104Cys | *-/-* | CS | 13 yr | MS | Rarely | VPA  (Sz - free) | Normal | Generalized spike-waves | Normal | IGE (JME) | [2] |
| 3-2 | M | c.311G>A/p.Phe104Cys | *-/-* | CS | 7. 5 yr | GTCS | Rarely | VPA, LTG  (Sz - free) | Normal | Generalized spike-waves generalized intermittent photic stimulation response | Normal | IGE  (GTCA) |  |
| 3-3 | F | c.311G>A/p.Phe104Cys | *-/-* | CS | 14 yr | GTCS | Rarely | NA (Sz - free) | Normal | Generalized spike-waves generalized intermittent photic stimulation response | Normal | IGE  (GTCA) |  |
| 4 | M | c.335G>A/p.Arg112Gln | *-/-* | *DN* | 11 mo | FS, GTCS, AS, fbTCS, MS | NA | NA | Moderate GDD | Generalized spike-wave | Normal | DEE  (DS) | [2] |
| 5 | F | c.335G>A/p.Arg112Gln | *-/-* | *DN* | 7 mo | GTCS, FS | Rarely | LEV (Sz - free) | GDD and Speech delay | Normal | Chiari I malfoemation | DEE  (DS-like) | [2] |
| 6-1 | M | c.335G>A/p.Arg112Gln | *-/-* | *DN* | 11 mo | FS, fbTCS, AS, SE, GTCS | NA | NA | Moderate GDD | Focal discharges | Calcified subependymal nodule in left lateral ventricle | DEE  (DS) | [2] |
| 6-2 |  | c.335G>A/p.Arg112Gln | *-/-* | *DN* | 10 mo | FS, GTCS, atonic | NA | VPA, CLB  (Sz - free) | Mild GDD and Speech delay | Symmetrical θ wave | Normal | DEE  (DS) | [5] |
| 7 | F | c.335G>A/p.Arg112Gln | *-/-* | *DN* | 8 mo | Clonic | NA | LEV (Sz - free) | Normal | Normal | Normal | IGE  (GTCA) | [2] |
| 8 | F | c.343A>G/p.Asn115Asp | *-/-* | *DN* | 6 mo | fbTCS, FS, GTCS | 3-5 times / 1 wk | NA | Moderate GDD, behavioral problems | Multifocal and bilateral spikes | Normal | DEE  (DS) | [2] |
| 9 | M | c.436C>A/p.Leu146Met | *-/-* | *DN* | 7 mo | FoS, fbTCS | every wks then mos | NA | Severe GDD | Multifocal paroxysmal activity | NA | DEE  (DS-like) | [2] |
| 10-1 | F | c.541C>T/p.Pro181Ser | *-/-* | *DN* | 12 yr | AS | NA | LTG, LEV (Sz - free for 13yr) | Mild-moderate GDD | NA | Normal | IGE  (JAE) | [3] |
| 10-2 | F | c.541C>T/p.Pro181Ser | *-/-* | *DN* | 8 yr | AS, atonic, GTCS | Daily | Refractory | Moderate GDD | Interictal:generalized polyspike waves and 2.5Hz spike waves  ictal: recorded absence seizures accompanied by generalized polyspikes and 2.5-3Hz spike wave activity. | Normal | DEE  (LGS) |  |
| 11 | F | c.640C>T/p.Arg214Cys | *-/-* | *DN* | 11 yr | FoS, GTCS, AS | 1 time / 1-2 mos | VPA, CZP (Remission) | GDD, ASD | Rhythmic generalized and bi-posterior quadrant spike and wave and polyspike and wave discharges which were time-locked with eyelid myoclonia | Periventricular leukomalacia and macrocephaly secondary to non-progressive ventriculomegaly | DEE | [4] |
| 12 | M | c.641G>A/p.Arg214His | *-/-* | *DN* | 8 mo | FS, GTCS | NA | Refractory | Severe GDD | Focal spike wave | NA | DEE  (DS) | [1] |
| 13 | M | c.641G>A/p.Arg214His | *-/-* | *DN* | 5 mo | FS, GTCS, FoS | NA | VPA, TPM (Remission) | Moderate GDD | Multifocal discharges, generalized spike-waves | NA | DEE  (DS) | [1] |
| 14 | F | c.641G>A/p.Arg214His | *-/-* | *DN* | 15 mo | FS, MS, GTCS, atonic, FoS | NA | Refractory | Severe GDD | Generalized spike-waves, bifrontal paroxysmal activity | Normal | DEE  (LGS) | [2] |
| 15 | F | c.751G>A/p.Gly251Ser | *-/-* | *DN* | 8 mo | FoS, GTCS, fbTCS, SE | NA | NA | Mild GDD | Normal | Normal | DEE  (DS) | [2] |
| 16 | M | c.752G>A/p.Gly251Asp | *-/-* | *DN* | 9 mo | FoS, | NA | VPA, LEV (Sz - free) | Speech delay | Slow background activity | NA | DEE | [2] |
| 17 | F | c.770C>G/p.Thr257Arg | *-/-* | *DN* | 4 mo | Spasms | NA | Refractory | Severe GDD and speech delay | Hypsarrhythmia, burst suppression, multifocal discharges | Normal | DEE  (WS) | [1] |
| 18 | F | c.778C> T/p.Pro260Ser | *-/-* | *DN* | 3 mo | Spasms, FoS, GTCS | NA | Refractory | Severe GDD | Hypsarrhythmia, burst suppression, multifocal discharges, generalized polyspike-wave | Normal | DEE  (WS) | [1] |
| 19 | F | c.779C>T/p.Pro260Leu | *-/-* | *DN* | 3.5 mo | Tonic, Spasms, MS | NA | VGB  (Remission) | Severe GDD, motor delay, speech delay and severe ID | Multifocal sharp-and-slow waves; hypsarrhythmia | Cerebral shrinkage due to ACTH therapy | DEE  (OS) | [5] |
| 20 | F | c.779C>T/p.Pro260Leu | *-/-* | *DN* | 1 mo | Spasms | NA | LTG  (Sz - free 3yr) | Severe GDD, motor delay, speech delay and severe ID | Diffuse spike-waves and polyspike-waves, hypsarrhythmia | Mild frontal lobe atrophy | DEE  (WS) | [5] |
| 21 | M | c.788T>C/p.Met263Thr | *-/-* | *DN* | 1 day | MS, Spasms | NA | Refractory | Severe GDD, motor delay, speech delay and severe ID | Multifocal sharp wave；hypsarrhythmia | Cerebral atrophy | DEE  (WS) | [5] |
| 22 | F | c.789G>C/p.Met263Ile | *-/-* | *DN* | 6 mo | Spasms | NA | ACTH  (Sz - free) | Severe GDD motor delay, speech delay and severe ID | Diffuse slow spike and waves, focal spikes at electrode on midline parietal region; hypsarrhythmia | Normal | DEE  (WS) | [5] |
| 23 | F | c.809T> C/p.Val270Ala | *-/-* | *DN* | 5 mo | FS, GTCS, FoS | NA | VPA, TPM, CZP (Remission) | Moderate GDD | Focal spike wave | Normal | DEE  (DS) | [1] |
| 24 | M | c.856G>T/p.Gly286Ter | *-/-* | Paternal | 6 yr | FoS | NA | OXC, TPM (Remission) | Moderate GDD | Generalized spike-waves | Normal | DEE | [1] |
| 25 | M | c.859G>T/p.Val287Leu | *-/-* | *DN* | 1 day | GTCS | NA | GBP  (Remission) | Severe GDD, motor delay, speech delay and severe ID | General slow background activity with multifocal spikes (7 mo); suppression burst (10 yr) | Cerebral, brain stem and cerebellar atrophy, thin corpus callosum | DEE | [5] |
| 26 | F | c.865A>C/p.Thr289Pro | *-/-* | *DN* | 1 day | Clonic, GTCS, FoS | Daily | Refractory | Severe GDD | Burst suppression, multifocal paroxysmal activity | Severe delayed myelination | DEE  (OS) | [2] |
| 27 | M | c.917A>C/p.Lys306Thr | *-/-* | *DN* | 8 mo | MS, GTCS | NA | Refractory | GDD, behavioral problems | Generalized spike-waves, bifrontal spike-wave, generalized intermittent photic stimulation response | Normal | DEE  (MAE) | [2] |
| 28 | M | c.917A>C/p.Lys306Thr | *-/-* | *DN* | 8 mo | AS, SE, FS, GTCS | NA | NA | Mild GDD | Generalized spike-wave, multifocal discharges, photoparoxysmal response | Normal | DEE  (DS) | [2] |
| 29-1 | NA | c.965C>A/p.Ala322Asp | *-/-* | CS | 13 yr | MS, GTCS | NA | NA | NA | Generalized spike-wave, polyspike-and-wave discharges | NA | IGE  (JME) | [6] |
| 29-2 | NA | c.965C>A/p.Ala322Asp | *-/-* | CS | 14 yr | MS, GTCS | NA | NA | NA | Generalized spike-wave, polyspike-and-wave discharges | NA | IGE  (JME) |  |
| 29-3 | NA | c.965C>A/p.Ala322Asp | *-/-* | CS | 12 yr | MS, GTCS | NA | NA | NA | Generalized spike-wave, polyspike-and-wave discharges | NA | IGE  (JME) |  |
| 29-4 | NA | c.965C>A/p.Ala322Asp | *-/-* | CS | 8 yr | MS, GTCS, AS | NA | NA | NA | Generalized spike-wave, polyspike-and-wave discharges | NA | IGE  (JME) |  |
| 29-5 | NA | c.965C>A/p.Ala322Asp | *-/-* | CS | 13 yr | MS, GTCS | NA | NA | NA | Generalized spike-wave, polyspike-and-wave discharges | NA | IGE  (JME) |  |
| 29-6 | NA | c.965C>A/p.Ala322Asp | *-/-* | CS | 16 yr | MS, GTCS, AS | NA | NA | NA | Generalized spike-wave, polyspike-and-wave discharges | NA | IGE  (JME) |  |
| 29-7 | NA | c.965C>A/p.Ala322Asp | *-/-* | CS | 13 yr | MS, GTCS, AS | NA | NA | NA | Generalized spike-wave, polyspike-and-wave discharges | NA | IGE  (JME) |  |
| 29-8 | NA | c.965C>A/p.Ala322Asp | *-/-* | CS | 5 yr | MS, GTCS, AS | NA | NA | NA | Generalized spike-wave, polyspike-and-wave discharges | NA | IGE  (JME) |  |
| 30 | M | c.995C>T/p.Ala332Val | *-/-* | *DN* | 2 mo | FoS | NA | TPM, LTG, PGB (Sz - free) | Severe GDD and motor delay | NA | A similar picture of a cerebral brain atrophy, especially in the frontal and temporal regions. | DEE | [7] |

F, female; M, male; ASD, autism spectrum disorder; AS, absence seizures; ACTH, adrenocorticotropic hormone; BZDs, benzodiazepines; DEX, dexamethasone; CNZ, clonazepam; CLB. clobazam; CZP, clonazepam; d, day; CS, Co-segregated; DN, De novo; DS. Dravet syndrome; DEE, developmental and epileptic encephalopathy; EEG, electroencephalography; EMA, eyelid myoclonia with or without absences; FS, febrile seizures; FoS, focal seizures; fbTCS, focal to bilateral tonic clonic seizure; GBP, gabapentin; GDD, global development delay; GTCA, generalized tonic-clonic seizures alone; GTCS, generalized tonic-clonic seizures; IGE, idiopathic generalized epilepsy; JAE, juvenile absence epilepsy; JME, juvenile myoclonic epilepsy; LGS, [Lennox-Gastaut syndrome](https://pubmed.ncbi.nlm.nih.gov/29124439/); LTG: lamotrigine; LEV, levetiracetam; MRI, magnetic resonance imaging; MS, myoclonic seizure; mo, month; MAE, myoclonic absence epilepsy; MAF, minor allele frequency; NA, not available; PE, partial epilepsy; OS, Ohtahara syndrome; OXC, oxcarbazepine; POLE, photosensitive occipital lobe epilepsy; PGB, pregabalin; SE, status epilepticus; Sz - free，seizure - free; TPM. topiramate; VGB, vigabatrin; VPA, valproate; WS, West syndrome; wk: week; yr, year; Pz, electrode on midline parietal region.

1. Zhang L, Liu X: **Clinical phenotype and genotype of children with GABAA receptor α1 subunit gene-related epilepsy**. *Frontiers in Neurology* 2022, **13**:941054.

2. Johannesen K, Marini C, Pfeffer S, Møller RS, Dorn T, Niturad CE, Gardella E, Weber Y, Søndergård M, Hjalgrim H: **Phenotypic spectrum of GABRA1: From generalized epilepsies to severe epileptic encephalopathies**. *Neurology* 2016, **87**(11):1140-1151.

3. Krenn M, Ernst M, Tomschik M, Treven M, Wagner M, Westphal DS, Meitinger T, Pataraia E, Zimprich F, Aull‐Watschinger S: **Phenotypic variability of GABRA1‐related epilepsy in monozygotic twins**. *Annals of Clinical and Translational Neurology* 2019, **6**(11):2317-2322.

4. Bai Y-F, Chiu M, Chan ES, Axerio-Cilies P, Lu J, Huh L, Connolly MB, Guella I, Farrer MJ, Xu Z-QD: **Pathophysiology of and therapeutic options for a GABRA1 variant linked to epileptic encephalopathy**. *Molecular brain* 2019, **12**:1-17.

5. Kodera H, Ohba C, Kato M, Maeda T, Araki K, Tajima D, Matsuo M, Hino‐Fukuyo N, Kohashi K, Ishiyama A: **De novo GABRA1 mutations in Ohtahara and West syndromes**. *Epilepsia* 2016, **57**(4):566-573.

6. Cossette P, Liu L, Brisebois K, Dong H, Lortie A, Vanasse M, Saint-Hilaire J-M, Carmant L, Verner A, Lu W-Y: **Mutation of GABRA1 in an autosomal dominant form of juvenile myoclonic epilepsy**. *Nature genetics* 2002, **31**(2):184-189.

7. Steudle F, Rehman S, Bampali K, Simeone X, Rona Z, Hauser E, Schmidt WM, Scholze P, Ernst M: **A novel de novo variant of GABRA1 causes increased sensitivity for GABA in vitro**. *Scientific Reports* 2020, **10**(1):2379.
